# Mid- and long-term risk of atrial fibrillation among breast cancer surgery survivors

**DOI:** 10.1186/s12916-024-03308-z

**Published:** 2024-02-28

**Authors:** Yong-Moon Mark Park, Wonyoung Jung, Yohwan Yeo, Sang Hyun Park, Michael G. Fradley, Sindhu J. Malapati, Tushar Tarun, Vinay Raj, Hong Seok Lee, Tasneem Z. Naqvi, Ronda S. Henry-Tillman, Jawahar L. Mehta, Mario Schootman, Benjamin C. Amick, Kyungdo Han, Dong Wook Shin

**Affiliations:** 1 Department of Epidemiology, Fay W. Boozman College of Public Health, University of Arkansas for Medical Sciences, Little Rock, AR USA; 2 Winthrop P. Rockefeller Cancer Institute, University of Arkansas for Medical Sciences, Little Rock, AR USA; 3 Department of Family Medicine / Obesity and Metabolic Health Center, College of Medicine, Hallym University Kangdong Sacred Heart Hospital, Seoul, Republic of Korea; 4 Department of Medicine, Sungkyunkwan University School of Medicine, Seoul, Republic of Korea; 5 Department of Family Medicine, College of Medicine, Hallym University Dongtan Sacred Heart Hospital, Hwaseong, Republic of Korea; 6 Department of Biostatistics, College of Medicine, The Catholic University of Korea, Seoul, Republic of Korea; 7 Cardio-Oncology Program, Division of Cardiology, Department of Internal Medicine, University of Pennsylvania, Philadelphia, PA USA; 8 Division of Medical Oncology, Department of Internal Medicine, University of Arkansas for Medical Sciences, Little Rock, AR USA; 9 Division of Cardiology, Department of Medicine, University of Arkansas for Medical Sciences, Little Rock, AR USA; 10 Department of Biology & Department of Math and Computer Science, University of Arkansas at Pine Bluff, Pine Bluff, AR USA; 11 Division of Cardiology, Sarver Heart Center, Banner University Medical Group, University of Arizona, Tucson, AZ USA; 12 Division of Echocardiography, Department of Cardiovascular Medicine, Mayo Clinic, Phoenix, AZ USA; 13 Division of Breast Surgical Oncology, Department of Surgery, University of Arkansas for Medical Sciences, Little Rock, AR USA; 14 Department of Medicine, College of Medicine, University of Arkansas for Medical Sciences, Little Rock, AR USA; 15 Department of Statistics and Actuarial Science, Soongsil University, 369 Sangdo-Ro, Dongjak-Gu, Seoul, 06978 Republic of Korea; 16 Department of Family Medicine and Supportive Care Center, Samsung Medical Center, Sungkyunkwan University School of Medicine, 81 Irwon-Ro, Gangnam-Gu, Seoul, 06351 Republic of Korea; 17 Department of Clinical Research Design & Evaluation, Samsung Advanced Institute for Health Science & Technology (SAIHST), Sungkyunkwan University, Seoul, 06351 Republic of Korea

**Keywords:** Atrial fibrillation, Breast cancer, Anthracyclines, Younger breast cancer survivors

## Abstract

**Background:**

The risk of incident atrial fibrillation (AF) among breast cancer survivors, especially for younger women, and cancer treatment effects on the association remain unclear. This study aimed to investigate the risk of AF among breast cancer survivors and evaluate the association by age group, length of follow-up, and cancer treatment.

**Methods:**

Using data from the Korean Health Insurance Service database (2010–2017), 113,232 women newly diagnosed with breast cancer (aged ≥ 18 years) without prior AF history who underwent breast cancer surgery were individually matched 1:5 by birth year to a sample female population without cancer (*n* = 566,160) (mean[SD] follow-up, 5.1[2.1] years). Sub-distribution hazard ratios (sHRs) and 95% confidence intervals (CIs) considering death as a competing risk were estimated, adjusting for sociodemographic factors and cardiovascular/non-cardiovascular comorbidities.

**Results:**

BCS had a slightly increased AF risk compared to their cancer-free counterparts (sHR 1.06; 95% CI 1.00–1.13), but the association disappeared over time. Younger BCS (age < 40 years) had more than a 2-fold increase in AF risk (sHR 2.79; 95% CI 1.98–3.94), with the association remaining similar over 5 years of follow-up. The increased risk was not observed among older BCS, especially those aged > 65 years. Use of anthracyclines was associated with increased AF risk among BCS (sHR 1.57; 95% CI 1.28–1.92), which was more robust in younger BCS (sHR 1.94; 95% CI 1.40–2.69 in those aged ≤ 50 years).

**Conclusions:**

Our findings suggest that younger BCS had an elevated risk of incident AF, regardless of the length of follow-up. Use of anthracyclines may be associated with increased mid-to-long-term AF risk among BCS.

**Graphical Abstract:**

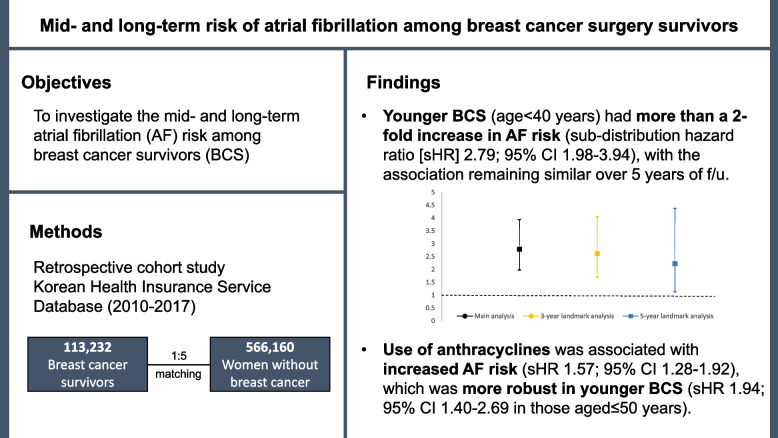

**Supplementary Information:**

The online version contains supplementary material available at 10.1186/s12916-024-03308-z.

## Background

Individuals treated for breast cancer may be at increased risk of developing atrial fibrillation (AF), which is one of the key drivers of cardiovascular (CV) complications such as myocardial ischemia, stroke, and heart failure [1, 2]. Proposed mechanisms for increased risk of AF among breast cancer survivors include shared common risk factors such as advancing age, alcohol consumption, smoking, and obesity, as well as cancer-related proinflammatory state and cardiotoxic cancer treatment [1–3]. Due to increased early detection and improved survival following breast cancer treatment [4], mid- to long-term CV complications have become a major concern of breast cancer survivors [2].

The evidence regarding the risk of AF among breast cancer survivors has been primarily derived from studies limited by relatively small numbers of patients evaluated over a short period [5]. Additionally, most studies have focused on women over 65 years [6, 7] or have been limited by the short-term follow-up (e.g., < 3 years) [6, 8], small numbers of incident AF events [9], use of prevalent breast cancer cases [8, 10], early-stage breast cancer [11], and failure to consider competing risks (e.g., death) [8, 12, 13], and comprehensive cancer treatment effects [12–14]. Consequently, the risk of AF among breast cancer survivors is unknown.

Breast cancer and CV events in women vary with age, particularly before and after menopause onset [2]. For instance, compared to those without cancer, women aged < 40 years with breast cancer are at more than a 3-fold increased risk of developing CV complications [15], and women diagnosed with breast cancer before age 50 years are at a higher risk of developing treatment-related CV complications [16]. However, few studies have investigated whether AF risk differs between younger and older breast cancer survivors [8], particularly in Asian women, whose breast cancer incidence peaks in the mid to late 40 s, which is remarkably different from that observed in Western countries [17]. Using the Korean National Health Insurance Service (NHIS) database, we evaluated incident AF among breast cancer survivors compared to women without cancer, particularly investigating the mid- to long-term risk of AF and the role of cancer treatment on this association by the age at the time of breast cancer diagnosis.

## Methods

### Data source and study setting

The NHIS is a public health insurance program providing mandatory universal coverage to 97% of the South Korean population. The NHIS database comprises sociodemographic and claims-based healthcare information such as medical procedures, diagnoses, prescriptions, outpatient visits, and hospital admission [18, 19]. The NHIS also operates biennial standardized health screening examinations [20], which include past medical history, lifestyle behaviors (smoking, drinking, and physical activity), anthropometric measurements, and laboratory tests for nonemployees aged ≥ 40 years and employees regardless of age. The NHIS database has been validated for epidemiological and clinical research [21, 22].

This study was approved by the Samsung Medical Center (Seoul, South Korea; SMC 2020-03-108) Institutional Review Board. All information used for analyses was anonymized and de-identified; therefore, informed consent was not required. The database is open to all researchers whose study protocols are approved by the official review committee.

### Study population

**Fig. 1:**
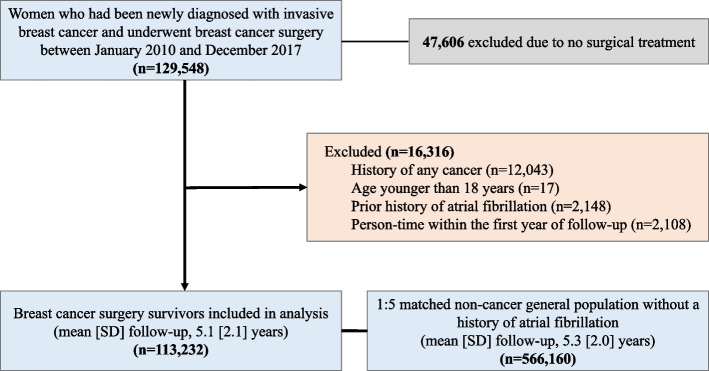
Participant flow diagram. After excluding participants meeting exclusion criteria, 113,232 subjects were included in the final analysis

We identified 129,548 women newly diagnosed with invasive breast cancer who had undergone breast cancer surgery between January 1, 2010, and December 31, 2017, based on both International Classification of Diseases, Tenth Revision (ICD-10) codes (C50) and cancer-specific insurance claim code (V193 code). This V code was used to ensure the accuracy of breast cancer diagnosis because it is a reimbursement code representing biopsy-confirmed cancers in South Korea [23]. We excluded those who did not undergo breast cancer surgery to avoid late-stage or aggressive breast cancer cases. Additionally, we excluded those who had a history of any cancer (*n* = 12,043), were younger than 18 years old (*n* = 17), or had a prior history of AF (*n* = 2,148). We further excluded person-time within the first year of follow-up to reduce the bias related to undetected AF present at baseline and the acute effect of breast cancer treatments on AF risk [1] (*n* = 2108). The resulting 113,232 breast cancer survivors were matched 1:5 to noncancer female general population with no prior history of AF (*n* = 566,160) from the general population based on birth year at baseline. Data recorded through December 31, 2020, were included in our analyses (Fig. 1).

### Measurements

The primary outcome was incident AF, based on ICD-10 codes of I48.0–I48.4, and I48.9. AF diagnosis using ICD-10 codes in the NHIS has been shown to be 94.1% accurate [24]. We defined individuals with AF as those who had a discharge diagnosis or visited an outpatient clinic more than twice to exclude those with transient AF and improve diagnostic accuracy [14, 25].

Breast cancer treatment information was based on the claims data within 1 year after the breast cancer diagnosis [23, 26]. Chemotherapy was defined as at least 1 treatment cycle of a chemotherapeutic agent (anthracyclines [epirubicin or doxorubicin], cyclophosphamide, and taxane-based regimens [docetaxel or paclitaxel]). Due to the reimbursement policy in the NHIS, most patients administered taxane-based regimens tended to take anthracyclines in the adjuvant setting, indicating that those given taxanes could also be included in a category of those who took anthracyclines [26]. Targeted therapy was defined as at least one treatment cycle of Herceptin (trastuzumab). Endocrine therapy was defined as treatment with tamoxifen or aromatase inhibitors (anastrozole, exemestane, and letrozole), categorizing the use of a specific regimen based on the initial prescription when there was a subsequent switching between medicines. Radiation therapy was defined as at least one local or regional treatment.

Comorbid medical conditions were assessed using past medical history data and clinical and pharmacy ICD-10 codes. Comorbidities included hypertension, type 2 diabetes, dyslipidemia, coronary heart disease (CHD), congestive heart failure, stroke, chronic kidney disease, and chronic obstructive pulmonary disease (COPD). Comorbidities of participants were identified based on laboratory measures, claims, and prescription information prior to the index date as follows: hypertension (ICD-10 codes [I10.x-I13.x and I15.x], or being on antihypertensive medication or having blood pressure ≥ 140/90 mmHg), diabetes mellitus (DM) (ICD-10 codes [E11.x-E14.x] with antidiabetic medications, or a fasting glucose level ≥ 126 mg/dL), dyslipidemia (ICD-10 code E78.x with lipid-lowering medication, or total cholesterol level ≥ 240 mg/dL), coronary heart disease (ICD-10 codes [I21.x-I22.x] during hospitalization), congestive heart failure (ICD codes [I50.x] for the first hospitalization), stroke (ICD-10 codes [I63.x-I64.x] during hospitalization, with claims for brain magnetic resonance imaging or brain computed tomography), chronic kidney disease (the glomerular filtration rate of < 60 mL/min/1.73 m^2^ as estimated by the Modification of Diet in Renal Disease equation), and chronic obstructive pulmonary disease (ICD-10 codes [J43.x-J44.x]). ICD-10 codes were also applied if recorded in at least two outpatient visits for coronary heart disease and stroke. In addition, the Charlson Comorbidity Index (CCI) was calculated based on ICD-10 codes. Income level was based on monthly health insurance premiums. Low-income status was defined as being in the lowest quartile of monthly health insurance premiums or being enrolled in the Medical Aid program. The geographic area of residence was dichotomized by rural and urban areas using the primary local authority districts (shi/gun/gu).

### Statistical analysis

The baseline characteristics are presented as the mean with standard deviations or numbers with percentages. Participant data were followed until the date of the first AF diagnosis, death, or end of the study, whichever occurred first. Person-time was calculated starting at 1 year after baseline. The crude AF incidence rate was assessed by dividing the number of events by the total number of person-years of follow-up presented as per 1000 person-years. Competing risk survival statistics considering death as a competing risk were used to calculate the cumulative incidence of AF [27]. The AF incidence between women with and without breast cancer was compared using the Gray-K test [28].

The Fine–Gray proportional sub-distribution hazards model was used to estimate sub-distribution hazard ratios (sHRs) and 95% confidence intervals (CIs) for AF incidence with death as a competing risk [29]. We applied the Fine–Gray model rather than the cause-specific hazards model because we aimed to evaluate the total effect of exposure (i.e., the presence of breast cancer in the total study population and cancer treatment in breast cancer survivors) on incident AF, not to determine the biological mechanism of the associations [30]. The proportional hazards assumption was assessed using Schoenfeld’s residuals, and no specific departure was observed. In addition to a crude model (Model 1), potential confounders were identified a priori based on a literature review; these included age at baseline (continuous variable), household income, and residential location, which were incorporated into Model 2 to account for sociodemographic characteristics in the association between the presence of breast cancer and AF risk. Model 3 further adjusted for the presence or absence of hypertension, type 2 diabetes, dyslipidemia, CHD, congestive heart failure, stroke, chronic kidney disease, and COPD to account for comorbidities. Then, we examined AF risk by different cancer treatment modalities, including anthracyclines, taxanes, trastuzumab, endocrine treatment, and radiation therapy among breast cancer survivors, further adjusting for the use of other cancer treatment modalities in Model 4. For instance, to examine the risk of AF, breast cancer survivors who underwent anthracycline treatment were compared with their counterparts who did not undergo anthracycline treatment. All analyses were stratified by age categories (18–39, 40–50, 51–65, or ≥ 66 years). Landmark analyses were performed using 2 landmark points (3 and 5 years after breast cancer diagnosis) to estimate AF risk in an unbiased way in individuals who were event-free at the landmark time and examine the long-term effects of breast cancer and its treatments on AF incidence [31].

As a sensitivity analysis, we included person-time within the first year of follow-up to capture any short-term CV consequences. In addition, using a subset of data comprising participants in the general health screening examination, we conducted sensitivity analyses among 72,560 women with and 292,468 without breast cancer to account for body mass index, smoking, alcohol consumption, and physical activity (see Additional file 1: Supplemental Methods for measurement details). A potential effect modification by income status, residential location, and other comorbidities was evaluated through stratified analysis and interaction testing using a likelihood ratio test. All statistical analyses were performed using SAS version 9.4 (SAS Institute Inc., Cary, NC, USA). The *P* values provided are two-sided, with the level of significance at 0.05.

## Results

### Baseline characteristics

Baseline characteristics are shown in Table 1, stratified by the presence or absence of breast cancer. The mean study population age was 51.6 years, and 10.8% of participants were less than 40 years old. Individuals surgically treated for breast cancer (hereafter referred to as breast cancer survivors) were more likely to have hypertension, type 2 diabetes, dyslipidemia, CHD, congestive heart failure, chronic kidney disease, and COPD than women without cancer. Among breast cancer survivors, the proportions of use in each treatment option were 61.0% for chemotherapy, 15.0% for target therapy, 61.8% for endocrine treatment, and 69.8% for radiation therapy. Breast cancer survivors were less likely to have low incomes or live in rural area. Overall, the prevalence of CV risk factors and comorbidities was higher in breast cancer survivors than in women without cancer in each age group (Additional file 2: Table S1). In addition, women with breast cancer aged 40 or over had a higher prevalence of CV risk factors and comorbidities compared to those with breast cancer younger than 40 years (Additional file 2: Table S2).

**Table 1:** Baseline characteristics of the study population and subgroup of general health examination participants

	Breast cancer	
	No (*N* = 566,160)	Yes (*N* = 113,232)
Age at baseline, mean (SD), years	51.6 (10.8)	51.6 (10.8)
Age group, years		
18–39	61,325 (10.8)	12,265 (10.8)
40–50	202,435 (35.8)	40,487 (35.8)
51–65	235,670 (41.6)	47,134 (41.6)
≥ 66	66,730 (11.8)	13,346 (11.8)
Comorbidity		
Hypertension	122,631 (21.7)	28,779 (25.4)
Type 2 diabetes	39,756 (7.0)	10,027 (8.9)
Dyslipidemia	100,734 (17.8)	23,068 (20.4)
Coronary heart disease	33,511 (5.9)	8732 (7.7)
Congestive heart failure	7670 (1.4)	3046 (2.7)
Stroke	12,177 (2.2)	2464 (2.2)
Chronic kidney disease	4444 (0.8)	1285 (1.1)
Chronic obstructive pulmonary disease	53,517 (9.5)	20,698 (18.3)
Charlson comorbidity index, mean (SD)	0.9 (1.2)	2.5 (1.3)
Cancer treatment type		
Chemotherapy, Yes		69,056 (61.0)
Anthracyclines		58,709 (85.0)
Cyclophosphamide		65,140 (94.3)
Fluorouracil		15,801 (22.9)
Taxane		36,417 (52.7)
Others^a^		7937 (11.5)
Trastuzumab, Yes		16,945 (15.0)
Endocrine therapy, Yes		77,925 (61.8)
Tamoxifen		49,574 (43.8)
Aromatase inhibitors		27,046 (23.9)
Both		1305 (1.2)
Radiation therapy, Yes		79,076 (69.8)
Income status, Low	140,488 (24.8)	25,507 (22.5)
Residential location, Urban	265,234 (46.8)	56,703 (50.1)
General health examination participants	No (*N* = 292,468)	Yes (*N* = 72,560)
Smoking, Ever	14,835 (5.1)	4216 (5.8)
Alcohol consumption (≥ 10 g), Yes	70,695 (24.2)	17,531 (24.2)
Regular physical activity^b^, Yes	54,062 (18.5)	13,330 (18.4)
Body mass index, mean (SD), kg/m^2^	23.6 (3.3)	23.6 (3.4)
Obesity (BMI ≥ 25 kg/m^2^)	86,548 (29.6)	22,264 (30.7)

Data are expressed as number (%) or mean (standard deviation) unless otherwise noted

^a^Methotrexate or cisplatin

^b^Regular physical activity was defined as at least 30 min of moderate physical activity for ≥ 5 days weekly or at least 20 min of strenuous physical activity ≥ 2 days weekly

### Risk of AF in breast cancer survivors compared to the general population by age group

**Fig. 2:**
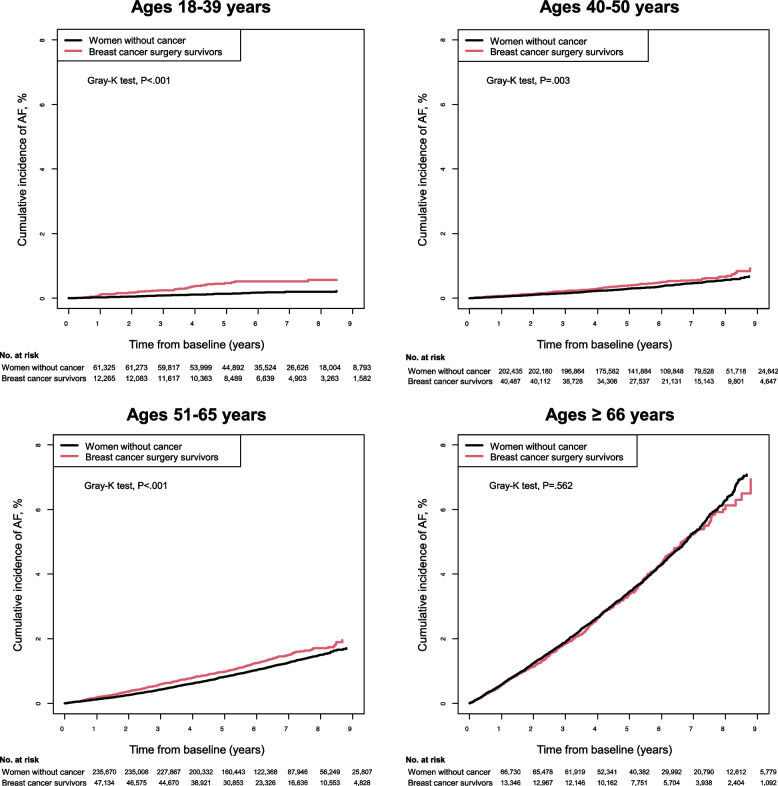
Cumulative incidence of atrial fibrillation (AF) by age group. Cumulative incidence function plots for atrial fibrillation (AF) in breast cancer surgery survivors display that breast cancer surgery survivors had a consistently higher incidence of AF compared to their age-matched noncancer female general population in those aged 18–39 (**a ***P* < 0.001), aged 40–50 (**b ***P* = 0.003), and aged 51–65 (**c ***P* < 0.001)

Risks of AF by age group are shown in Fig. 2 and Table 2. A total of 1166 (1.0%) incident AF cases were identified at least 1 year after baseline during a mean (SD) of 5.1 (2.1) years of follow-up in breast cancer survivors. Sub-distribution hazard (sub-distribution hazard is hereafter referred to as risk) of AF development was 6% higher in breast cancer survivors than those without cancer (Model 3: sHR 1.06, 95% CI 1.00–1.13) after adjusting for potential confounders. Associations differed by age groups (*P* for interaction < 0.001). Younger women (18–39 years) with breast cancer exhibited a 2.79-fold increased AF risk (sHR 2.79, 95% CI 1.98–3.94), whereas older women (≥ 66 years) with breast cancer exhibited a decreased AF risk (sHR 0.90, 95% CI 0.81–0.99), compared with those without cancer. In the landmark analysis, AF risk was attenuated over time, and there was no overall association with AF over 5 years after breast cancer diagnosis. However, younger women with breast cancer exhibited a sustained twofold increased risk in those aged < 40 years and at least a 30% increased AF risk in those aged ≤ 50 years during follow-up (Additional file 2: Table S3). In contrast, the inverse association tended to persist in those aged > 65 years (*P* for interaction for age group in both landmark analyses < 0.05).

**Table 2:** Adjusted sub-distribution hazard ratios for developing atrial fibrillation in breast cancer surgery survivors compared to the noncancer general population by age categories

	**Age group**		Subjects (*N*)	Case (*n*)	IR per 1000 person-years	Model 1 (Crude) sHR (95% CI)	Model 2 sHR (95% CI)	Model 3 sHR (95% CI)
***Main analysis***	***All ages***	Noncancer	566,160	5,271	1.76	1 (Ref.)	1 (Ref.)	1 (Ref.)
		Breast cancer	113,232	1,166	2.01	1.14 (1.07–1.22)	1.15 (1.08–1.23)	1.06 (1.00–1.13)
	***18*–*39***	Noncancer	61,325	91	0.27	1 (Ref.)	1 (Ref.)	1 (Ref.)
		Breast cancer	12,265	51	0.78	2.94 (2.09–4.15)	2.95 (2.10–4.16)	2.79 (1.98–3.94)
	***40*–*50***	Noncancer	202,435	684	0.63	1 (Ref.)	1 (Ref.)	1 (Ref.)
		Breast cancer	40,487	171	0.81	1.28 (1.09–1.52)	1.29 (1.09–1.53)	1.22 (1.03–1.45)
	***51*–*65***	Noncancer	235,670	2,123	1.71	1 (Ref.)	1 (Ref.)	1 (Ref.)
		Breast cancer	47,134	497	2.06	1.21 (1.10–1.33)	1.22 (1.10–1.34)	1.13 (1.03–1.25)
	**≥ *66***	Noncancer	66,730	2,373	7.31	1 (Ref.)	1 (Ref.)	1 (Ref.)
		Breast cancer	13,346	447	7.08	0.97 (0.88–1.07)	0.98 (0.89–1.09)	0.90 (0.81–0.99)
*P* for interaction						< 0.001	< 0.001	< 0.001
***3*–*year landmark analysis***	***All ages***	Noncancer	546,453	3,609	1.94	1 (Ref.)	1 (Ref.)	1 (Ref.)
		Breast cancer	107,153	777	2.17	1.12 (1.04–1.21)	1.13 (1.05–1.22)	1.05 (0.97–1.14)
	***18*–*39***	Noncancer	59,817	60	0.28	1 (Ref.)	1 (Ref.)	1 (Ref.)
		Breast cancer	11,617	31	0.76	2.75 (1.78–4.24)	2.76 (1.79–4.26)	2.62 (1.70–4.05)
	***40*–*50***	Noncancer	196,863	472	0.69	1 (Ref.)	1 (Ref.)	1 (Ref.)
		Breast cancer	38,727	117	0.89	1.28 (1.05–1.57)	1.29 (1.06–1.58)	1.23 (1.00–1.50)
	***51*–*65***	Noncancer	227,856	1,516	1.97	1 (Ref.)	1 (Ref.)	1 (Ref.)
		Breast cancer	44,665	328	2.22	1.13 (1.00–1.27)	1.14 (1.01–1.28)	1.06 (0.94–1.20)
	**≥ *66***	Noncancer	61,917	1,561	8.04	1 (Ref.)	1 (Ref.)	1 (Ref.)
		Breast cancer	12,144	301	8.06	1.00 (0.89–1.14)	1.02 (0.90–1.15)	0.93 (0.82–1.05)
*P* for interaction						< 0.001	< 0.001	< 0.001
***5-year landmark analysis***	***All ages***	Noncancer	546,453	1,918	2.11	1 (Ref.)	1 (Ref.)	1 (Ref.)
		Breast cancer	107,153	388	2.25	1.07 (0.96–1.19)	1.08 (0.96–1.19)	1.00 (0.90–1.12)
	***18*–*39***	Noncancer	59,817	28	0.25	1 (Ref.)	1 (Ref.)	1 (Ref.)
		Breast cancer	11,617	12	0.59	2.32 (1.18–4.56)	2.33 (1.18–4.57)	2.22 (1.13–4.37)
	***40*–*50***	Noncancer	196,863	265	0.79	1 (Ref.)	1 (Ref.)	1 (Ref.)
		Breast cancer	38,727	65	1.01	1.28 (0.98–1.68)	1.29 (0.98–1.69)	1.23 (0.94–1.62)
	***51*–*65***	Noncancer	227,856	812	2.18	1 (Ref.)	1 (Ref.)	1 (Ref.)
		Breast cancer	44,665	163	2.31	1.06 (0.90–1.25)	1.06 (0.90–1.26)	1.00 (0.84–1.18)
	**≥ *66***	Noncancer	61,917	813	9.08	1 (Ref.)	1 (Ref.)	1 (Ref.)
		Breast cancer	12,144	148	8.70	0.96 (0.80–1.14)	0.96 (0.81–1.15)	0.89 (0.74–1.06)
*P* for interaction						0.041	0.041	0.024

Landmark analysis was conducted to estimate AF risk in individuals who were event-free at specific time points (landmark time), 3 and 5 years post-breast cancer diagnosis, respectively

Model 2: adjusted for age, income status, and residential location

Model 3: adjusted for Model 2 + hypertension, type 2 diabetes, dyslipidemia, coronary heart disease, congestive heart failure, chronic kidney disease, and chronic obstructive pulmonary disease

*IR* Incidence rate, *PYs* Person-years, *sHR* Sub-distribution hazard ratio, *CI* Confidence interval

### Risk of AF by treatment modalities

Risks of AF by cancer treatment modalities among breast cancer survivors are shown in Table 3. Breast cancer survivors who received anthracyclines had a 57% higher AF risk compared with those who did not (Model 4: sHR 1.57, 95% CI 1.28–1.92), whereas tamoxifen users had a 19% lower AF risk compared with those who did not receive endocrine treatment (sHR 0.81, 95% CI 0.70–0.95). Breast cancer survivors who received taxane-based chemotherapy had increased AF risk compared with those who did not receive taxane-based chemotherapy (Model 3: sHR 1.20, 95% CI 1.06–1.35), but the association became nonsignificant after further adjusting for other cancer treatment modalities (Model 4: sHR 0.84, 95% CI 0.69–1.02). Other cancer therapies, including trastuzumab, aromatase inhibitors, and radiation treatment, were not associated with AF incidence among breast cancer survivors. In the landmark analyses, breast cancer survivors who used anthracyclines showed persistent increased AF risk over the follow-up period compared with those who did not use anthracyclines. In contrast, the inverse association between tamoxifen use and AF risk tended to disappear.

**Table 3:** Adjusted sub-distribution hazard ratios for developing atrial fibrillation by cancer treatment type among breast cancer surgery survivors

	**Treatment type**		Subjects (*N*)	Case (*n*)	IR per 1000 PYs	Model 1 (Crude) sHR (95% CI)	Model 2 sHR (95% CI)	Model 3 sHR (95% CI)	Model 4 sHR (95% CI)
***Main analysis***	***Anthracyclines***	No	54,523	591	2.14	1 (Ref.)	1 (Ref.)	1 (Ref.)	1 (Ref.)
		Yes	58,709	575	1.89	0.88 (0.79–0.98)	1.39 (1.23–1.58)	1.39 (1.23–1.57)	1.57 (1.28–1.92)
	***Taxane***	No	48,092	530	2.17	1 (Ref.)	1 (Ref.)	1 (Ref.)	1 (Ref.)
		Yes	65,140	636	1.89	0.87 (0.77–0.97)	1.22 (1.08–1.37)	1.20 (1.06–1.35)	0.84 (0.69–1.02)
	***Trastuzumab***	No	96,287	996	2.00	1(Ref.)	1 (Ref.)	1 (Ref.)	1 (Ref.)
		Yes	16,945	170	2.04	1.03 (0.87–1.21)	1.15 (0.97–1.35)	1.11 (0.94–1.30)	0.96 (0.80–1.14)
	***Endocrine therapy***	No	35,307	386	2.19	1 (Ref.)	1 (Ref.)	1 (Ref.)	1 (Ref.)
		Tamoxifen	49,574	294	1.12	0.51 (0.44–0.59)	0.79 (0.68–0.92)	0.78 (0.67–0.92)	0.81 (0.70–0.95)
		AIs	27,046	471	3.46	1.58 (1.38–1.81)	1.01 (0.88–1.15)	1.00 (0.87–1.15)	1.00 (0.87–1.15)
		Both	1305	15	2.23	1.02 (0.61–1.70)	0.70 (0.42–1.17)	0.66 (0.40–1.11)	0.68 (0.41–1.14)
	***Radiation therapy***	No	34,156	433	2.52	1 (Ref.)	1 (Ref.)	1 (Ref.)	1 (Ref.)
		Yes	79,076	733	1.79	0.71 (0.63–0.80)	1.01 (0.89–1.14)	1.02 (0.90–1.16)	0.98 (0.87–1.12)
***3-year landmark analysis***	***Anthracyclines***	No	52,036	390	2.32	1 (Ref.)	1 (Ref.)	1 (Ref.)	1 (Ref.)
		Yes	55,117	387	2.04	0.88 (0.76–1.01)	1.41 (1.22–1.64)	1.40 (1.21–1.63)	1.48 (1.16–1.90)
	***Taxane***	No	45,695	345	2.31	1 (Ref.)	1(Ref.)	1 (Ref.)	1 (Ref.)
		Yes	61,458	432	2.07	0.90 (0.78–1.03)	1.27 (1.10–1.48)	1.25 (1.08–1.45)	0.91 (0.72–1.16)
	***Trastuzumab***	No	91,190	663	2.15	1 (Ref.)	1 (Ref.)	1 (Ref.)	1 (Ref.)
		Yes	15,963	114	2.28	1.07 (0.88–1.30)	1.19 (0.98–1.46)	1.16 (0.95–1.41)	0.99 (0.80–1.23)
	***Endocrine therapy***	No	32,452	252	2.34	1 (Ref.)	1 (Ref.)	1 (Ref.)	1 (Ref.)
		Tamoxifen	47,839	208	1.27	0.54 (0.45–0.65)	0.85 (0.70–1.03)	0.85 (0.70–1.02)	0.89 (0.74–1.08)
		AIs	25,633	308	3.72	1.59 (1.35–1.88)	0.99 (0.84–1.18)	0.99 (0.83–1.17)	0.99 (0.83–1.17)
		Both	1229	9	2.16	0.92 (0.47–1.79)	0.62 (0.32–1.21)	0.59 (0.30–1.14)	0.61 (0.31–1.18)
	***Radiation therapy***	No	31,842	283	2.70	1 (Ref.)	1 (Ref.)	1 (Ref.)	1 (Ref.)
		Yes	75,311	494	1.95	0.72 (0.62–0.84)	1.04 (0.89–1.21)	1.05 (0.90–1.22)	1.01 (0.87–1.18)
***5-year landmark analysis***	***Anthracyclines***	No	34,664	183	2.32	1 (Ref.)	1 (Ref.)	1 (Ref.)	1 (Ref.)
		Yes	39,965	205	2.20	0.95 (0.78–1.16)	1.57 (1.27–1.94)	1.56 (1.26–1.93)	1.56 (1.10–2.21)
	***Taxane***	No	31,154	160	2.28	1 (Ref.)	1 (Ref.)	1 (Ref.)	1 (Ref.)
		Yes	43,475	228	2.24	0.98 (0.80–1.20)	1.43 (1.16–1.76)	1.40 (1.14–1.73)	1.02 (0.72–1.43)
	***Trastuzumab***	No	64,089	332	2.22	1 (Ref.)	1 (Ref.)	1 (Ref.)	1 (Ref.)
		Yes	10,540	56	2.45	1.11 (0.84–1.47)	1.24 (0.93–1.65)	1.20 (0.90–1.59)	1.01 (0.75–1.37)
	***Endocrine therapy***	No	22,325	115	2.23	1 (Ref.)	1 (Ref.)	1 (Ref.)	1 (Ref.)
		Tamoxifen	34,191	116	1.46	0.65 (0.50–0.84)	1.04 (0.80–1.35)	1.04 (0.80–1.35)	1.13 (0.86–1.48)
		AIs	17,253	152	3.89	1.74 (1.37–2.22)	1.07 (0.83–1.36)	1.06 (0.83–1.36)	1.08 (0.84–1.38)
		Both	860	5	2.49	NA	NA	NA	NA
	***Radiation therapy***	No	21,464	144	2.86	1 (Ref.)	1 (Ref.)	1 (Ref.)	1 (Ref.)
		Yes	53,165	244	2.00	0.70 (0.57–0.86)	1.02 (0.82–1.26)	1.03 (0.83–1.28)	0.98 (0.79–1.22)

Model 2: adjusted for age, income status, and residential location

Model 3: adjusted for Model 2 + hypertension, type 2 diabetes, dyslipidemia, coronary heart disease, congestive heart failure, chronic kidney disease, and chronic obstructive pulmonary disease

Model 4: adjusted for Model 3 + use of anthracyclines, taxane, trastuzumab, endocrine therapy, and radiation therapy

*IR* Incidence rate, *PYs* Person-years, *sHR* Sub-distribution hazard ratio, *CI* Confidence interval, *AIs* Aromatase inhibitors, *NA* Not applicable

Risks of AF according to cancer treatment modalities and age group among breast cancer survivors are shown in Table 4. Breast cancer survivors who used anthracyclines had increased AF risk compared with those who did not use anthracyclines in all age groups, with a stronger association among younger women aged ≤ 50 years. The inverse association between tamoxifen use and AF risk also tended to be clearer among younger women aged ≤ 50 years (Additional file 2: Table S4). In the landmark analyses, the use of anthracyclines tended to be consistently associated with increased AF risk in each age group over the follow-up. In contrast, the inverse association between tamoxifen use and AF risk was no longer significant (Additional file 2: Table S5).

**Table 4:** Adjusted sub-distribution hazard ratios for developing atrial fibrillation by cancer treatment type among breast cancer surgery survivors by age categories

**Treatment type**	Age 18–39			Age 40–50			Age 51–65			Age ≥ 66		
	C/S	IR	sHR (95% CI)	C/S	IR	sHR (95% CI)	C/S	IR	sHR (95% CI)	C/S	IR	sHR (95% CI)
***Anthracyclines***												
No	9/4532	0.38	1 (Ref.)	59/18,346	0.62	1 (Ref.)	195/21,802	1.75	1 (Ref.)	328/9843	7.07	1 (Ref.)
Yes	42/7733	1.02	3.07 (1.47–6.42)	112 /22,141	0.95	1.69 (1.18–2.40)	302/25,332	2.33	1.53 (1.20–1.93)	119/3503	7.10	1.51 (1.15–1.98)
***Taxane***												
No	15/4426	0.64	1 (Ref.)	56/16,694	0.65	1 (Ref.)	170/18,796	1.77	1 (Ref.)	289/8176	7.47	1 (Ref.)
Yes	36/7839	0.86	0.92 (0.49–1.71)	115/23,793	0.92	0.93 (0.65–1.33)	327/28,338	2.26	0.85 (0.67–1.09)	158/5170	6.45	0.81 (0.63–1.03)
***Trastuzumab***												
No	44/10,465	0.78	1 (Ref.)	142/35,100	0.77	1 (Ref.)	410/38,765	2.05	1 (Ref.)	400/11,957	7.02	1 (Ref.)
Yes	7/1800	0.78	0.85 (0.38–1.89)	29/5387	1.08	1.16 (0.78–1.74)	87/8369	2.12	0.88 (0.69–1.12)	47/1389	7.57	1.05 (0.77–1.44)
***Endocrine therapy***												
No	24/4462	1.04	1 (Ref.)	53/10,629	0.99	1 (Ref.)	168/15,998	2.10	1 (Ref.)	141/4218	7.34	1 (Ref.)
Tamoxifen	27/7741	0.65	0.59 (0.34–1.02)	106/28,566	0.70	0.72 (0.52–1.00)	91/11,024	1.57	0.94 (0.72–1.21)	70/2243	6.32	0.82 (0.61–1.09)
AIs	0/35	0	NA	8/1149	1.27	1.01 (0.48–2.13)	233/19,253	2.37	1.04 (0.85–1.27)	230/6609	7.30	1.03 (0.84–1.27)
Both	0/27	0	NA	4/143	5.15	NA	5/859	1.12	NA	6/276	4.40	0.57 (0.25–1.28)
***Radiation therapy***												
No	12/3485	0.67	1 (Ref.)	42/11,160	0.73	1 (Ref.)	144/13,091	2.17	1 (Ref.)	235/6420	7.81	1 (Ref.)
Yes	39/8780	0.83	1.18 (0.62–2.25)	129/29,327	0.83	1.11 (0.79–1.58)	353/34,043	2.02	0.96 (0.79–1.17)	212/6926	6.41	0.99 (0.82–1.20)

Hazard ratios were adjusted for age, income status, residential location, hypertension, type 2 diabetes, dyslipidemia, coronary heart disease, congestive heart failure, chronic kidney disease, chronic obstructive pulmonary disease, and history of anthracyclines, taxane, trastuzumab, endocrine therapy, and radiation therapy

*C/S* The number of cases/the number of subjects, *IR* Incidence rate (described as per 1000 person-years), *sHR* Sub-distribution hazard ratio, *CI* Confidence interval, *AIs* Aromatase inhibitors, *NA* Not applicable

### Stratified analyses and sensitivity analyses

In stratified analyses, increased AF risk was observed only for those without CHD (*P* for interaction = 0.04) or those without COPD (*P* for interaction = 0.06) among women with breast cancer of all ages (Additional file 2: Table S6). When we included person-time within the first year of follow-up, the associations tended to be stronger, particularly in younger breast cancer survivors (aged ≤ 50 years) (Additional file 2: Table S7). In contrast, meaningful changes were not observed in risks of AF by cancer treatment modalities among breast cancer survivors (Additional file 2: Table S8). The results were not materially changed in analyses limited to participants in the NHIS health examinations where body mass index, smoking, alcohol consumption, and physical activity were further adjusted for (Additional file 2: Tables S9 and S10).

## Discussion

In this nationwide population-based cohort study, younger breast cancer survivors had increased AF risk compared to an age-matched sample of females with no cancer history from the general population; the strength of this association remained persistent over 5 years of follow-up. Breast cancer survivors aged < 40 years had a more than twofold increased AF risk during follow-up; this elevated risk was not observed in older breast cancer survivors, particularly those aged > 65 years. Among breast cancer survivors, those treated with anthracyclines had a more pronounced AF risk than those not exposed to this chemotherapeutic. The association was strongest in breast cancer survivors aged ≤ 50 years, and remained persistent over the follow-up period.

Newly developed AF in individuals with cancer may have an adverse effect on prognosis. In a study using the US Surveillance, Epidemiology, and End Results Medicare registry data, old women aged > 65 years with breast cancer who developed incident AF within the first 30 days of breast cancer diagnosis were 3 times more likely to die of a CV condition within 1 year compared with those without incident AF [6]. In a Taiwanese retrospective cohort study, individuals with cancer who developed new-onset AF had an increased risk of thromboembolism and heart failure, although all-cause mortality was not elevated [32]. In contrast, new-onset AF in those with lymphoma was positively associated with both acute heart failure and all-cause mortality [33]. In addition, there was a higher risk of bleeding in patients with cancer and AF than in those without AF [34].

While we initially observed an overall positive association between breast cancer and the risk of developing AF, it was marginal and became null over the follow-up period. These results are similar to previous studies, which were unable to show a strong association between breast cancer and incident AF compared to other types of cancer [13, 14]. The strength of the association with the risk of incident AF also decreased over time, with the highest risk occurring in the first year of diagnosis [6, 7, 11, 13, 14], which was also observed in our study. This transient, increased risk of developing AF shortly after breast cancer diagnosis might be due to surgical and/or medical cancer therapy, autonomic nervous system imbalance, pre-existing chronic inflammatory state, cancer-related comorbidities, or the combination of these conditions [1]. To assess the mid-to-long-term risk of AF among breast cancer survivors, we excluded AF incidence in the first year of follow-up to mitigate the effects of these potential confounding factors.

In our study, younger survivors particularly aged < 40 years showed a stronger association with incident AF than older breast cancer survivors. The increased risk remained persistent over 5 years of the follow-up after adjusting for cardiometabolic comorbidities. A previous study reported that breast cancer survivors < 60 years had a stronger association with incident AF than older breast cancer survivors [8]. It is unclear why the association differs by age group. In our stratified analyses, among those without CV risk factors or CVD at baseline, breast cancer survivors had a higher AF risk compared to the general population. This suggests that AF incidence between older breast cancer survivors and the general population might not be different due to the increased prevalence of CV risk factors and comorbid CVD as women age. However, the prevalence of CV risk factors and comorbidities in women aged > 40 years was still higher in breast cancer survivors compared to the general population in our study. The other speculation is that younger patients receive more intensive, cardiotoxic treatments than older patients [8]. Tanenbaum et al. reported that younger cancer survivors are more likely to undergo screening for cardiovascular risk factors, compared to younger people without cancer [35]. However, given our findings of the overall 1% incidence of AF in this study cohort, the low incidence rate demands careful interpretation for clinical implications. Regular cardiac surveillance or monitoring for AF may be warranted, although further research is needed to establish a specific prevention strategy [36].

We observed a positive association between the use of anthracyclines and subsequent increased AF risk after accounting for use of other cancer treatment modalities. After adjusting for other cancer treatment modalities, there was no significant association between the use of taxanes and AF occurrence, suggesting that the observed unadjusted association was likely driven by the concomitant use of anthracyclines [26]. This is consistent with prior findings from a recent study of the World Health Organization dataset VigiBase of more than 130 countries, which reported that those who developed AF were more likely to use anthracyclines, adjusting for the use of other anticancer medications [37]. In a meta-analysis conducted in 2013, subclinical manifestations of cardiac toxicity were identified in 18% of the patients treated with an anthracycline after an average follow-up of 9 years, which was higher compared to 6% developed clinical cardiac toxicity [38]. Patients with more aggressive forms of breast cancer, such as triple-negative breast cancer, might be more likely to receive anthracyclines, which could make them more susceptible to AF [39]. Anthracyclines, such as doxorubicin, are known for their cardiotoxic effects, primarily due to the generation of reactive oxygen species leading to oxidative stress and myocardial damage [40]. This oxidative stress can trigger structural and electrical remodeling of the atrial myocardium, which is a known substrate for AF development [41]. Moreover, anthracyclines can induce cellular apoptosis and disrupt cardiac calcium metabolism, further contributing to AF incidence [42]. In contrast, our findings differ from those of previous studies from the US [6] and Canada [11], where anthracycline use was not associated with increased AF risk, possibly because of different study population characteristics such as racial/ethnic differences [43]. However, racial/ethnic differences in cardiotoxicity is understudied and further studies are warranted to confirm the observed finding [44]. Moreover, younger patients or those with fewer pre-existing cardiac risk factors might be preferentially selected for these treatments, impacting the observed association between anthracyclines and the incidence of AF. An inverse association between tamoxifen use and AF risk among younger breast cancer survivors aged ≤ 50 years tended to diminish gradually with increasing follow-up duration. This finding suggests that benefit of tamoxifen use may be limited to reduced risk of early CV outcomes in breast cancer survivors [45].

Given the limitations of claims data, we could not provide clinical details, including cancer stage, cardiac imaging, pathological results, surgery type, and chemotherapy dosage. In addition, misclassification and unmeasured confounding may exist as a result of limitations inherent to claims data. However, we investigated the mid- to long-term risk of AF after a breast cancer diagnosis and its surgical/medical treatments using data from a large sample size of 113,232 women newly diagnosed with breast cancer. We did not include some established AF risk factors such as obesity and lifestyle characteristics in our main analysis. However, we did perform various sensitivity analyses including further adjustment for body mass index, smoking, alcohol consumption, and physical activity to address this issue. In addition, there might be false positive findings due to a lack of correction for multiple comparisons. By employing the Fine–Gray model, our sub-distribution hazard ratio estimates may exhibit biases when compared to cause-specific hazard ratios [30]. Lastly, our definition of AF, requiring at least two diagnoses of AF for inclusion, may bias our outcome towards more severe cases, potentially underrepresenting milder or transient forms of this condition.

## Conclusions

In conclusion, young breast cancer surgery survivors, specifically those aged < 40 years and those treated with anthracyclines, may be associated with increased mid- to long-term risk of AF. Our findings underscore the need for increased awareness of AF risk in this population.

### Supplementary Information

**Additional file 1.** Supplementary Methods.

**Additional file 2: Table S1.** Baseline cardiovascular risk and comorbidities by age. **Table S2.** Cardiovascular risk factors in < 40 vs. ≥ 40 age groups. **Table S3.** Hazard ratios for AF in young breast cancer survivors. **Table S4.** AF risk by treatment in young survivors. **Table S5.** AF risk by age and treatment at 3- and 5-year marks. **Table S6.** AF risk in survivors vs. general population by factors. **Table S7.** Sensitivity analysis of AF risk by age after 1 year. **Table S8.** Treatment-based AF risk by age after 1 year. **Table S9.** AF risk in survivors vs. general population: Health screening subset. **Table S10.** Treatment-based AF risk: Health screening subset.

## Data Availability

The data will be made available upon request and approval of a proposal by the National Health Insurance Service Database.

## Abbreviations

AF: Atrial fibrillation
CHD: Coronary heart disease
CI: Confidence interval
COPD: Chronic obstructive pulmonary disease
CV: Cardiovascular
DM: Diabetes mellitus
NHIS: National Health Insurance Service
SD: Standard deviation
sHR: Sub-distribution hazard ratio
