# Supplementary material for: Mid- and long-term risk of atrial fibrillation among breast cancer surgery survivors
Source: BMC Med. 2024 Feb 28;22:88. doi: 10.1186/s12916-024-03308-z (PMC10903065; doi:10.1186/s12916-024-03308-z)
Supplement: Supplementary file 1 — Additional file 1. Supplementary Methods. [file 12916_2024_3308_MOESM1_ESM.docx]

**SUPPLEMENTAL METHODS**

**Measurement of Covariates in participants in the general health screening examination**

Information on lifestyle-related factors was obtained using the self-administered questionnaires at enrollment. Smoking status was categorized as never or ever. Alcohol consumption was categorized into non-drinking or mild drinking (<10 g/day) and moderate to heavy drinking (≥10 g/day). For physical activity, regular exercise was defined to be at least 30 minutes of moderate physical activity for ≥5 days weekly or at least 20 minutes of strenuous physical activity ≥2 days weekly.

The anthropometric and clinical characteristics were assessed by trained personnel during the health screening examination. Body mass index (BMI) was calculated by weight in kilograms divided by the square of height in meters (kg/m^2^). Obesity was defined as BMI ≥25kg/m^2^. ^1^

1. Seo MH, Lee WY, Kim SS, et al: 2018 Korean Society for the Study of Obesity Guideline for the Management of Obesity in Korea. J Obes Metab Syndr 28:40-45, 2019
