# Supplementary material for: Mid- and long-term risk of atrial fibrillation among breast cancer surgery survivors
Source: BMC Med. 2024 Feb 28;22:88. doi: 10.1186/s12916-024-03308-z (PMC10903065; doi:10.1186/s12916-024-03308-z)
Supplement: Supplementary file 2 — Additional file 2: Table S1. Baseline cardiovascular risk and comorbidities by age. Table S2. Cardiovascular risk factors in < 40 vs. ≥ 40 age groups. Table S3. Hazard ratios for AF in young breast cancer survivors. Table S4. AF risk by treatment in young survivors. Table S5. AF risk by age and treatment at 3- and 5-year marks. Table S6. AF risk in survivors vs. general population by factors. Table S7. Sensitivity analysis of AF risk by age after 1 year. Table S8. Treatment-based AF risk by age after 1 year. Table S9. AF risk in survivors vs. general population: Health screening subset. Table S10. Treatment-based AF risk: Health screening subset. [file 12916_2024_3308_MOESM2_ESM.docx]

**Table S1.** Comparison of baseline cardiovascular risk factors and comorbidities by age group

|  | Aged 18-39 | | | Aged 18-50 | | | Aged ≥ 51 | | |
| --- | --- | --- | --- | --- | --- | --- | --- | --- | --- |
|  | Noncancer  (n=61,325) | Breast cancer  (n=12,265) | P value | Noncancer  (n=263,760) | Breast cancer  (n=52,752) | P value | Noncancer  (n=302,400) | Breast cancer  (n=60,480) | P value |
| Income status, low | 12,698 (20.7) | 2,239 (18.3) | <.001 | 62,961 (23.9) | 11,162 (21.2) | <.001 | 77,527 (25.6) | 14,345 (23.7) | <.001 |
| Residential location, Urban | 29,358 (47.9) | 6,061 (49.4) | <.001 | 124,104 (47.1) | 25,930 (49.2) | <.001 | 141,130 (46.7) | 30,773 (50.9) | <.001 |
| Hypertension | 1,166 (1.9) | 522 (4.3) | <.001 | 17,950 (6.8) | 4,846 (9.2) | <.001 | 104,681 (34.6) | 23,933 (39.6) | <.001 |
| Type 2 diabetes | 504 (0.8) | 165 (1.4) | <.001 | 5,730 (2.2) | 1,527 (2.9) | <.001 | 34,026 (11.3) | 8,500 (14.1) | <.001 |
| Dyslipidemia | 832 (1.4) | 366 (3.0) | <.001 | 13,406 (5.1) | 3,521 (6.7) | <.001 | 87,328 (28.9) | 19,547 (32.3) | <.001 |
| Coronary heart disease | 653 (1.1) | 320 (2.6) | <.001 | 5,054 (1.9) | 1,816 (3.4) | <.001 | 28,457 (9.4) | 6,916 (11.4) | <.001 |
| Congestive heart failure | 123 (0.2) | 191 (1.6) | <.001 | 11,009 (4.2) | 832 (1.6) | <.001 | 6,661 (2.2) | 2,214 (3.7) | <.001 |
| Chronic kidney disease | 170 (0.3) | 51 (0.4) | 0.104 | 998 (0.4) | 324 (0.6) | <.001 | 3,446 (1.1) | 961 (1.6) | <.001 |
| COPD | 3,619 (5.9) | 1,804 (14.7) | <.001 | 16,356 (6.2) | 7,382 (14.0) | <.001 | 37,161 (12.3) | 13,316 (22.0) | <.001 |

COPD; chronic obstructive pulmonary disease.

**Table S2.** Comparison of baseline cardiovascular risk factors and comorbidities by age group (aged <40 vs. ≥40 years)

|  | Aged 18-39 | | | Aged ≥ 40 | | | P * | P ** |
| --- | --- | --- | --- | --- | --- | --- | --- | --- |
|  | Noncancer | Breast cancer | P value | Noncancer | Breast cancer | P value |  |  |
| Income status, low | 12,698 (20.7) | 2,239 (18.3) | <.001 | 127,790 (25.3) | 23,268 (23.0) | <.001 | 0.221 | 0.226 |
| Residential location, Urban | 29,358 (47.9) | 6,061 (49.4) | 0.002 | 235,876 (46.7) | 50,642 (50.2) | <.001 | <.001 | <.001 |
| Hypertension | 1,166 (1.9) | 522 (4.3) | <.001 | 121,465 (24.1) | 28,257 (28.0) | <.001 | <.001 | <.001 |
| Type 2 diabetes | 504 (0.8) | 165 (1.4) | <.001 | 39,252 (7.8) | 9,862 (9.8) | <.001 | 0.006 | 0.006 |
| Dyslipidemia | 832 (1.4) | 366 (3.0) | <.001 | 99,902 (19.8) | 22,702 (22.5) | <.001 | <.001 | <.001 |
| Coronary heart disease | 653 (1.1) | 320 (2.6) | <.001 | 32,858 (6.5) | 8,412 (8.3) | <.001 | <.001 | <.001 |
| Congestive heart failure | 123 (0.2) | 191 (1.6) | <.001 | 17,547 (3.5) | 2,855 (2.8) | <.001 | <.001 | <.001 |
| Chronic kidney disease | 170 (0.3) | 51 (0.4) | 0.010 | 4,274 (0.8) | 1,234 (1.2) | <.001 | 0.826 | 0.826 |
| COPD | 3,619 (5.9) | 1,804 (14.7) | <.001 | 49,898 (9.9) | 18,894 (18.7) | <.001 | <.001 | <.001 |

COPD; chronic obstructive pulmonary disease.

P* : Breslow-Day test

P** : P for interaction

**Table S3.** Adjusted sub-distribution hazard ratios for developing atrial fibrillation in breast cancer surgery survivors compared to the noncancer general population (aged 18-50 years)

|  | | Subjects (N) | Case (n) | IR per 1,000 person-years | Model 1 (Crude)  HR (95% CI) | Model 2  sHR (95% CI) | Model 3  sHR (95% CI) |
| --- | --- | --- | --- | --- | --- | --- | --- |
| ***Main analysis*** | Noncancer | 263,760 | 775 | 0.54 | 1(Ref.) | 1(Ref.) | 1(Ref.) |
|  | Breast cancer | 52,752 | 222 | 0.80 | 1.48 (1.27-1.72) | 1.48 (1.28-1.72) | 1.40 (1.21-1.63) |
| ***3-year landmark analysis*** | Noncancer | 256,680 | 532 | 0.59 | 1(Ref.) | 1(Ref.) | 1(Ref.) |
|  | Breast cancer | 50,344 | 148 | 0.86 | 1.45 (1.21-1.74) | 1.45 (1.21-1.74) | 1.38 (1.15-1.66) |
| ***5-year landmark analysis*** | Noncancer | 256,680 | 293 | 0.66 | 1(Ref.) | 1(Ref.) | 1(Ref.) |
|  | Breast cancer | 50,344 | 77 | 0.91 | 1.39 (1.08-1.78) | 1.39 (1.08-1.78) | 1.33 (1.03-1.71) |

IR, incidence rate; HR, hazard ratio; sHR, sub-distribution hazard ratio; CI, confidence interval.

Landmark analysis was conducted to estimate AF risk in individuals who were event-free at specific time points (landmark time), three and five years post-breast cancer diagnosis, respectively.

Model 2: adjusted for age, income status, and residential location.

Model 3: adjusted for Model 2 + hypertension, type 2 diabetes, dyslipidemia, coronary heart disease, congestive heart failure, chronic kidney disease, and chronic obstructive pulmonary disease.

**Table S4.** Adjusted sub-distribution hazard ratios for developing atrial fibrillation by cancer treatment type among breast cancer surgery survivors aged 18-50 years

|  | | ***Main analysis*** | | | | ***3-year landmark analysis*** | | | | ***5-year landmark analysis*** | | | |
| --- | --- | --- | --- | --- | --- | --- | --- | --- | --- | --- | --- | --- | --- |
|  | | Subjects (N) | Case (n) | IR | sHR (95% CI) | Subjects (N) | Case (n) | IR | sHR (95% CI) | Subjects (N) | Case (n) | IR | sHR (95% CI) |
| ***Anthracycline*** | No | 22,878 | 68 | 0.57 | 1 (Ref.) | 22,117 | 45 | 0.62 | 1(Ref.) | 15,059 | 21 | 0.61 | 1(Ref.) |
|  | Yes | 29,874 | 154 | 0.97 | 1.94 (1.40-2.69) | 28,227 | 103 | 1.03 | 1.79 (1.20-2.68) | 20,967 | 56 | 1.12 | 1.86 (1.05-3.29) |
| ***Taxane*** | No | 21,120 | 71 | 0.65 | 1 (Ref.) | 20,274 | 44 | 0.65 | 1(Ref.) | 14,161 | 19 | 0.59 | 1(Ref.) |
|  | Yes | 31,632 | 151 | 0.90 | 0.94 (0.68-1.31) | 30,070 | 104 | 0.99 | 1.08 (0.72-1.62) | 21,865 | 58 | 1.11 | 1.31 (0.72-2.37) |
| ***Trastuzumab*** | No | 45,565 | 186 | 0.77 | 1 (Ref.) | 43,531 | 125 | 0.83 | 1(Ref.) | 31,416 | 63 | 0.85 | 1(Ref.) |
|  | Yes | 7,187 | 36 | 1.00 | 1.09 (0.76-1.57) | 6,813 | 23 | 1.06 | 1.08 (0.69-1.69) | 4,610 | 14 | 1.38 | 1.37 (0.76-2.45) |
| ***Endocrine treatment*** | No | 15,091 | 77 | 1.01 | 1 (Ref.) | 13,903 | 45 | 0.95 | 1(Ref.) | 9,785 | 24 | 1.04 | 1(Ref.) |
|  | Tamoxifen | 36,307 | 133 | 0.69 | 0.66 (0.50-0.87) | 35,151 | 95 | 0.79 | 0.79 (0.56-1.13) | 25,303 | 50 | 0.85 | 0.78 (0.48-1.27) |
|  | AIs | 1,184 | 8 | 1.24 | 0.85 (0.41-1.76) | 1,137 | 7 | 1.71 | 1.21 (0.55-2.70) | 832 | 3 | 1.43 | 0.88 (0.27-2.93) |
|  | Both | 170 | 4 | 4.52 | NA | 153 | 1 | 1.79 | 1.41 (0.19-10.21) | 106 | 0 | 0 | NA |
| ***Radiation treatment*** | No | 14,645 | 54 | 0.72 | 1 (Ref.) | 13,831 | 35 | 0.76 | 1(Ref.) | 9,455 | 13 | 0.58 | 1(Ref.) |
|  | Yes | 38,107 | 168 | 0.83 | 1.13 (0.83-1.54) | 36,513 | 113 | 0.89 | 1.15 (0.79-1.68) | 26,571 | 64 | 1.03 | 1.69 (0.93-3.07) |

IR, incidence rate (described as per 1,000 person-years); HR, hazard ratio; sHR, sub-distribution hazard ratio; CI, confidence interval; AIs, aromatase inhibitors.

Hazard ratios were adjusted for income, area of residence, hypertension, diabetes mellitus, dyslipidemia, coronary heart disease, congestive heart failure, chronic kidney disease, chronic obstructive pulmonary disease, history of anthracyclines, taxane, trastuzumab, endocrine therapy, and radiation therapy.

**Table S5.** Landmark analyses at the 3- and 5-year landmark points for adjusted sub-distribution hazard ratios for developing atrial fibrillation by age categories and cancer treatment type among breast cancer surgery survivors

|  | | ***3-year landmark analysis*** | | | | ***5-year landmark analysis*** | | | | |
| --- | --- | --- | --- | --- | --- | --- | --- | --- | --- | --- |
| ***Age 18-39*** | | Subjects (N) | Case (n) | IR | sHR (95% CI) | Subjects (N) | Case (n) | IR | | sHR (95% CI) |
| ***Anthracycline*** | No | 4,365 | 6 | 0.40 | 1(Ref.) | 3,044 | 4 | | 0.55 | 1(Ref.) |
|  | Yes | 7,252 | 25 | 0.96 | 2.50 (1.00-6.23) | 5,445 | 8 | | 0.61 | 1.07 (0.31-3.68) |
| ***Taxane*** | No | 4,215 | 9 | 0.61 | 1(Ref.) | 3,025 | 6 | | 0.83 | 1(Ref.) |
|  | Yes | 7,402 | 22 | 0.84 | 0.98 (0.44,2.18) | 5,464 | 6 | | 0.45 | 0.39 (0.12-1.24) |
| ***Trastuzumab*** | No | 9,927 | 27 | 0.76 | 1(Ref.) | 7,309 | 12 | | 0.67 | 1(Ref.) |
|  | Yes | 1,690 | 4 | 0.73 | 0.823(0.288,2.356) | 1,180 | 0 | | 0 | NA |
| ***Endocrine treatment*** | No | 4,080 | 12 | 0.83 | 1(Ref.) | 2,965 | 6 | | 0.83 | 1(Ref.) |
|  | Tamoxifen | 7,485 | 19 | 0.72 | 0.82 (0.40-1.68) | 5,497 | 6 | | 0.46 | 0.52 (0.17-1.61) |
|  | AIs | 32 | 0 | 0 | NA | 18 | 0 | | 0 | NA |
|  | Both | 20 | 0 | 0 | NA | 9 | 0 | | 0 | NA |
| ***Radiation treatment*** | No | 3,257 | 5 | 0.45 | 1(Ref.) | 2238 | 2 | | 0.37 | 1(Ref.) |
|  | Yes | 8,360 | 26 | 0.87 | 1.84(0.71,4.79) | 6251 | 10 | | 0.66 | 1.71 (0.37-7.79) |
| ***Age 40-50*** | | Subjects (N) | Case (n) | IR | sHR (95% CI) | Subjects (N) | Case (n) | IR | | sHR (95% CI) |
| ***Anthracycline*** | No | 17,752 | 39 | 0.67 | 1(Ref.) | 12,015 | 17 | | 0.62 | 1(Ref.) |
|  | Yes | 20,975 | 78 | 1.06 | 1.62 (1.05-2.49) | 15,522 | 48 | | 1.30 | 2.02 (1.09-3.74) |
| ***Taxane*** | No | 16,059 | 35 | 0.66 | 1(Ref.) | 11,136 | 13 | | 0.52 | 1(Ref.) |
|  | Yes | 22,668 | 82 | 1.04 | 1.11 (0.71-1.73) | 16,401 | 52 | | 1.33 | 1.80 (0.92-3.53) |
| ***Trastuzumab*** | No | 33,604 | 98 | 0.85 | 1(Ref.) | 24,107 | 51 | | 0.90 | 1(Ref.) |
|  | Yes | 5,123 | 19 | 1.17 | 1.15 (0.70-1.89) | 3,430 | 14 | | 1.86 | 1.70 (0.94-3.09) |
| ***Endocrine treatment*** | No | 9,823 | 33 | 1.00 | 1(Ref.) | 6,820 | 18 | | 1.14 | 1(Ref.) |
|  | Tamoxifen | 27,666 | 76 | 0.80 | 0.82 (0.54-1.23) | 19,806 | 44 | | 0.96 | 0.86 (0.50-1.50) |
|  | AIs | 1,105 | 7 | 1.74 | 1.38 (0.61-3.12) | 814 | 3 | | 1.45 | 0.98 (0.29-3.34) |
|  | Both | 133 | 1 | 2.00 | 1.66 (0.23-12.11) | 97 | 0 | | 0 | NA |
| ***Radiation treatment*** | No | 10,574 | 30 | 0.85 | 1(Ref.) | 7,217 | 11 | | 0.65 | 1(Ref.) |
|  | Yes | 28,153 | 87 | 0.90 | 1.03 (0.68-1.56) | 20,320 | 54 | | 1.15 | 1.68 (0.88-3.22) |
| ***Age 51-65*** | | Subjects (N) | Case (n) | IR | sHR (95% CI) | Subjects (N) | Case (n) | IR | | sHR (95% CI) |
| ***Anthracycline*** | No | 20,936 | 120 | 1.76 | 1(Ref.) | 13,965 | 53 | | 1.66 | 1(Ref.) |
|  | Yes | 23,729 | 208 | 2.60 | 1.59 (1.18-2.13) | 16,887 | 110 | | 2.84 | 1.75 (1.15-2.66) |
| ***Taxane*** | No | 17,976 | 103 | 1.75 | 1(Ref.) | 12,227 | 42 | | 1.53 | 1(Ref.) |
|  | Yes | 26,689 | 225 | 2.52 | 1.03 (0.76-1.40) | 18,625 | 121 | | 2.80 | 1.33 (0.84-2.09) |
| ***Trastuzumab*** | No | 36,766 | 269 | 2.18 | 1(Ref.) | 25,672 | 136 | | 2.29 | 1(Ref.) |
|  | Yes | 7,899 | 59 | 2.40 | 0.94 (0.70-1.25) | 5,180 | 27 | | 2.40 | 0.88 (0.58-1.35) |
| ***Endocrine treatment*** | No | 14,849 | 112 | 2.28 | 1(Ref.) | 10,226 | 48 | | 2.05 | 1(Ref.) |
|  | Tamoxifen | 10,631 | 60 | 1.66 | 0.92 (0.67-1.26) | 7,482 | 36 | | 2.06 | 1.34 (0.87-2.09) |
|  | AIs | 18,370 | 152 | 2.53 | 1.02 (0.79-1.30) | 12,562 | 76 | | 2.66 | 1.20 (0.83-1.73) |
|  | Both | 815 | 4 | 1.44 | 0.62 (0.23-1.67) | 582 | 3 | | 2.26 | 1.13 (0.35-3.64) |
| ***Radiation treatment*** | No | 12,263 | 93 | 2.28 | 1(Ref.) | 8,338 | 50 | | 2.53 | 1(Ref.) |
|  | Yes | 32,402 | 235 | 2.19 | 0.99 (0.78-1.27) | 22,514 | 113 | | 2.22 | 0.90 (0.64-1.26) |
| ***Age≥66*** | | Subjects (N) | Case (n) | IR | sHR (95% CI) | Subjects (N) | Case (n) | IR | | sHR (95% CI) |
| ***Anthracycline*** | No | 8,983 | 225 | 8.23 | 1(Ref.) | 5,640 | 109 | | 8.81 | 1(Ref.) |
|  | Yes | 3,161 | 76 | 7.59 | 1.25 (0.90-1.75) | 2,111 | 39 | | 8.40 | 1.23 (0.77-1.97) |
| ***Taxane*** | No | 7,445 | 198 | 8.65 | 1(Ref.) | 4,766 | 99 | | 9.53 | 1(Ref.) |
|  | Yes | 4,699 | 103 | 7.12 | 0.79 (0.58-1.06) | 2,985 | 49 | | 7.38 | 0.75(0.49-1.15) |
| ***Trastuzumab*** | No | 10,893 | 269 | 7.95 | 1(Ref.) | 7,001 | 133 | | 8.56 | 1(Ref.) |
|  | Yes | 1,251 | 32 | 9.09 | 1.08 (0.74-1.58) | 750 | 15 | | 10.13 | 1.04 (0.60-1.82) |
| ***Endocrine treatment*** | No | 3,700 | 95 | 8.49 | 1(Ref.) | 2,314 | 43 | | 8.54 | 1(Ref.) |
|  | Tamoxifen | 2,057 | 53 | 7.88 | 0.92 (0.65-1.29) | 1,406 | 30 | | 9.45 | 1.17 (0.73-1.88) |
|  | AIs | 6,126 | 149 | 8.00 | 0.99 (0.76-1.28) | 3,859 | 73 | | 8.66 | 1.11 (0.76-1.63) |
|  | Both | 261 | 4 | 4.84 | 0.54 (0.20-1.46) | 172 | 2 | | 5.32 | 0.60 (0.14-2.46) |
| ***Radiation treatment*** | No | 5,748 | 155 | 8.73 | 1(Ref.) | 3,671 | 81 | | 9.90 | 1(Ref.) |
|  | Yes | 6,396 | 146 | 7.45 | 1.01 (0.80-1.28) | 4,080 | 67 | | 7.58 | 0.89 (0.64-1.25) |

IR, incidence rate (described as per 1,000 person-years); sHR, sub-distribution hazard ratio; CI, confidence interval; AIs, aromatase inhibitors.

Hazard ratios were adjusted for income, area of residence, hypertension, diabetes mellitus, dyslipidemia, coronary heart disease, congestive heart failure, chronic kidney disease, chronic obstructive pulmonary disease, and use of anthracyclines, taxane, trastuzumab, endocrine therapy, and radiation therapy.

**Table S6.** Adjusted sub-distribution hazard ratios for developing atrial fibrillation in breast cancer surgery survivors compared to the noncancer general population stratified by selected factors

|  | | | All ages | | | | Aged 18-50 years | | | |
| --- | --- | --- | --- | --- | --- | --- | --- | --- | --- | --- |
|  |  |  | Subjects (N) | Case (n) | IR per  1,000 PYs | sHR (95% CI) | Subjects (N) | Case (n) | IR per  1,000 PYs | sHR (95% CI) |
| ***Income*** | *High* | Noncancer | 425,672 | 3,799 | 1.69 | 1(Ref.) | 200,799 | 551 | 0.51 | 1(Ref.) |
|  |  | Breast cancer | 87,725 | 884 | 1.96 | 1.07 (0.99-1.15) | 41,590 | 164 | 0.75 | 1.36 (1.14-1.62) |
|  | *Low* | Noncancer | 140,488 | 1,472 | 2.00 | 1(Ref.) | 62,961 | 224 | 0.66 | 1(Ref.) |
|  |  | Breast cancer | 25,507 | 282 | 2.19 | 1.05 (0.92-1.19) | 11,162 | 58 | 1.00 | 1.39 (1.04-1.86) |
|  | P for interaction | | 0.809 | | | | 0.891 | | | |
| ***Residence*** | *Urban* | Noncancer | 265,234 | 2,204 | 1.57 | 1(Ref.) | 124,104 | 325 | 0.48 | 1(Ref.) |
|  |  | Breast cancer | 56,703 | 575 | 1.96 | 1.12 (1.02-1.23) | 25,930 | 95 | 0.69 | 1.34 (1.07-1.69) |
|  | *Rural* | Noncancer | 300,926 | 3,067 | 1.94 | 1(Ref.) | 139,656 | 450 | 0.60 | 1(Ref.) |
|  |  | Breast cancer | 56,529 | 591 | 2.06 | 1.02 (0.93-1.11) | 26,822 | 127 | 0.91 | 1.39 (1.14-1.69) |
|  | P for interaction | | 0.138 | | | | 0.827 | | | |
| ***Hypertension*** | *No* | Noncancer | 443,529 | 2,348 | 1.00 | 1(Ref.) | 245,810 | 618 | 0.47 | 1(Ref.) |
|  |  | Breast cancer | 84,453 | 491 | 1.12 | 1.14 (1.03-1.25) | 47,906 | 167 | 0.66 | 1.37 (1.16-1.63) |
|  | *Yes* | Noncancer | 122,631 | 2,923 | 4.62 | 1(Ref.) | 17,950 | 157 | 1.60 | 1(Ref.) |
|  |  | Breast cancer | 28,779 | 675 | 4.72 | 1.02 (0.93-1.11) | 4,846 | 55 | 2.18 | 1.35 (0.99-1.84) |
|  | P for interaction | | 0.087 | | | | 0.939 | | | |
| ***DM*** | *No* | Noncancer | 526,404 | 4,318 | 1.55 | 1(Ref.) | 258,030 | 729 | 0.52 | 1(Ref.) |
|  |  | Breast cancer | 103,205 | 920 | 1.73 | 1.08 (1.01-1.16) | 51,225 | 208 | 0.77 | 1.37 (1.18-1.61) |
|  | *Yes* | Noncancer | 39,756 | 953 | 4.79 | 1(Ref.) | 5,730 | 46 | 1.52 | 1(Ref.) |
|  |  | Breast cancer | 10,027 | 246 | 5.06 | 1.00 (0.87-1.15) | 1,527 | 14 | 1.81 | 1.28 (0.71-2.34) |
|  | P for interaction | | 0.302 | | | | 0.829 | | | |
| ***Dyslipidemia*** | *No* | Noncancer | 465,426 | 3,496 | 1.41 | 1(Ref.) | 250,354 | 684 | 0.51 | 1(Ref.) |
|  |  | Breast cancer | 90,164 | 751 | 1.60 | 1.10 (1.01-1.19) | 49,231 | 200 | 0.77 | 1.42 (1.21-1.66) |
|  | *Yes* | Noncancer | 100,734 | 1,775 | 3.55 | 1(Ref.) | 13,406 | 91 | 1.30 | 1(Ref.) |
|  |  | Breast cancer | 23,068 | 415 | 3.69 | 1.01 (0.91-1.12) | 3,521 | 22 | 1.22 | 1.04 (0.65-1.65) |
|  | P for interaction | | 0.224 | | | | 0.211 | | | |
| ***CHD*** | *No* | Noncancer | 532,649 | 4,122 | 1.46 | 1(Ref.) | 258,706 | 706 | 0.51 | 1(Ref.) |
|  |  | Breast cancer | 104,500 | 906 | 1.68 | 1.10 (1.03-1.19) | 50,936 | 200 | 0.75 | 1.41 (1.20-1.65) |
|  | *Yes* | Noncancer | 33,511 | 1,149 | 6.75 | 1(Ref.) | 5,054 | 69 | 2.53 | 1(Ref.) |
|  |  | Breast cancer | 8,732 | 260 | 6.06 | 0.94 (0.82-1.08) | 1,816 | 22 | 2.39 | 1.05 (0.65-1.71) |
|  | P for interaction | | **0.042** | | | | 0.261 | | | |
| ***CHF*** | *No* | Noncancer | 558,490 | 4,854 | 1.64 | 1(Ref.) | 262,751 | 752 | 0.53 | 1(Ref.) |
|  |  | Breast cancer | 110,186 | 1,057 | 1.86 | 1.07 (1,10-1.44) | 51,920 | 212 | 0.78 | 1.39 (1.19-1.63) |
|  | *Yes* | Noncancer | 7,670 | 417 | 12.04 | 1(Ref.) | 1,009 | 23 | 4.76 | 1(Ref.) |
|  |  | Breast cancer | 3,046 | 109 | 8.63 | 1.01 (0.82-1.25) | 832 | 10 | 2.90 | 0.91 (0.43-1.92) |
|  | P for interaction | | 0.624 | | | | 0.276 | | | |
| ***CKD*** | *No* | Noncancer | 561,716 | 5,075 | 1.71 | 1(Ref.) | 262,762 | 760 | 0.54 | 1(Ref.) |
|  |  | Breast cancer | 111,947 | 1,107 | 1.92 | 1.06 (1.00-1.14) | 52,428 | 218 | 0.79 | 1.38 (1.18-1.61) |
|  | *Yes* | Noncancer | 4,444 | 196 | 9.66 | 1(Ref.) | 998 | 15 | 2.98 | 1(Ref.) |
|  |  | Breast cancer | 1,285 | 59 | 10.57 | 1.07 (0.80-1.43) | 324 | 4 | 2.60 | 0.92 (0.30-2.77) |
|  | P for interaction | | 0.975 | | | | 0.476 | | | |
| ***COPD*** | *No* | Noncancer | 512,643 | 4,290 | 1.58 | 1(Ref.) | 247,404 | 710 | 0.53 | 1(Ref.) |
|  |  | Breast cancer | 92,534 | 842 | 1.77 | 1.11 (1.03-1.19) | 45,370 | 179 | 0.75 | 1.35 (1.14-1.59) |
|  | *Yes* | Noncancer | 53,517 | 981 | 3.53 | 1(Ref.) | 16,356 | 65 | 0.74 | 1(Ref.) |
|  |  | Breast cancer | 20,698 | 324 | 3.05 | 0.96 (0.85-1.09) | 7,382 | 43 | 1.09 | 1.51 (1.02-2.22) |
|  | P for interaction | | **0.055** | | | | 0.596 | | | |

IR, incidence rate; PYs, person-years; HR, hazard ratio; sHR, sub-distribution hazard ratio; CI, confidence interval; HTN, hypertension; DM, diabetes mellitus; DL, dyslipidemia; CHD, coronary heart disease; CHF, congestive heart failure; CKD, chronic kidney disease; COPD; chronic obstructive pulmonary disease.

Each model was adjusted for age, income, area of residence, hypertension, diabetes mellitus, dyslipidemia, coronary heart disease, congestive heart failure, chronic kidney disease, and chronic obstructive pulmonary disease, but not for the covariate used in stratified analysis.

**Table S7.** Adjusted sub-distribution hazard ratios for developing atrial fibrillation in breast cancer surgery survivors compared to the noncancer general population by age categories: **A sensitivity analysis after including person-time within the first year of follow-up**

|  | | | sHR (95% CI) |
| --- | --- | --- | --- |
| ***Age group*** | ***All ages*** | Noncancer | 1(Ref.) |
|  |  | Breast cancer | 1.60(1.52-1.69) |
|  | ***18-39*** | Noncancer | 1(Ref.) |
|  |  | Breast cancer | 6.84(5.18-9.03) |
|  | ***40-50*** | Noncancer | 1(Ref.) |
|  |  | Breast cancer | 2.81(2.48-3.19) |
|  | ***51-65*** | Noncancer | 1(Ref.) |
|  |  | Breast cancer | 1.71(1.58-1.85) |
|  | ***≥66*** | Noncancer | 1(Ref.) |
|  |  | Breast cancer | 1.01(0.92-1.11) |
| *P* for interaction | | | <0.001 |

sHR, sub-distribution hazard ratio; CI, confidence interval.

Adjusted for age, income, area of residence, hypertension, diabetes mellitus, dyslipidemia, coronary heart disease, congestive heart failure, chronic kidney disease, and chronic obstructive pulmonary disease.

**Table S8.** Adjusted sub-distribution hazard ratios for developing atrial fibrillation by cancer treatment type among breast cancer surgery survivors by age categories: **A sensitivity analysis after including person-time within the first year of follow-up**

|  | | All ages | Age 18-39 | Age 40-50 | Age 51-65 | Age ≥66 |
| --- | --- | --- | --- | --- | --- | --- |
|  |  | sHR (95% CI) | sHR (95% CI) | sHR (95% CI) | sHR (95% CI) | sHR (95% CI) |
| ***Anthracyclines*** | No | 1(Ref.) | 1(Ref.) | 1(Ref.) | 1(Ref.) | 1(Ref.) |
|  | Yes | 1.72(1.48-2.00) | 2.24 (1.29-4.51) | 1.86 (1.30-2.65) | 1.81 (1.44-2.28) | 1.51 (1.15-1.98) |
| ***Taxane*** | No | 1(Ref.) | 1(Ref.) | 1(Ref.) | 1(Ref.) | 1(Ref.) |
|  | Yes | 0.81(0.70-0.94) | 0.68 (0.39-1.18) | 0.90 (0.63-1.28) | 0.79 (0.63-1.00) | 0.90 (0.69-1.16) |
| ***Trastuzumab*** | No | 1(Ref.) | 1(Ref.) | 1(Ref.) | 1(Ref.) | 1(Ref.) |
|  | Yes | 0.97(0.86-1.10) | 0.92 (0.58-1.43) | 1.04 (0.80-1.35) | 0.91 (0.76-1.09) | 1.14 (0.87-1.49) |
| ***Endocrine therapy*** | No | 1(Ref.) | 1(Ref.) | 1(Ref.) | 1(Ref.) | 1(Ref.) |
|  | Tamoxifen | 0.89(0.79-1.00) | 0.90 (0.63-1.27) | 0.92 (0.73-1.16) | 0.83 (0.67-1.03) | 0.88 (0.67-1.16) |
|  | AIs | 0.98(0.88-1.10) | 1.97 (0.27-14.2) | 0.88 (0.51-1.53) | 0.92 (0.79-1.08) | 1.13 (0.93-1.38) |
|  | Both | 0.89(0.62-1.29) | N/A | 2.62 (1.06-6.44) | 0.82 (0.41-1.26) | 0.91 (0.50-1.63) |
| ***Radiation treatment*** | No | 1(Ref.) | 1(Ref.) | 1(Ref.) | 1(Ref.) | 1(Ref.) |
|  | Yes | 0.89(0.81-0.98) | 1.04 (0.69-1.54) | 0.78 (0.63-0.96) | 0.93 (0.79-1.08) | 0.96 (0.81-1.14) |

sHR, sub-distribution hazard ratio; CI, confidence interval; AIs, aromatase inhibitors.

Adjusted for age, income, area of residence, hypertension, diabetes mellitus, dyslipidemia, coronary heart disease, congestive heart failure, chronic kidney disease, and chronic obstructive pulmonary disease, history of anthracycline, taxane, trastuzumab, endocrine treatment, and radiation treatment.

**Table S9.** Adjusted sub-distribution hazard ratios for developing atrial fibrillation in breast cancer surgery survivors compared to the noncancer general population by age categories: **A subset analysis based on data comprising participants in the general health screening examination**

| **Age group** | | Subjects (N) | Case (n) | IR per  1,000 PYs | Model 1 (Crude)  HR (95% CI) | Model 2  sHR (95% CI) | Model 3  sHR (95% CI) | Model 4  sHR (95% CI) |
| --- | --- | --- | --- | --- | --- | --- | --- | --- |
| ***All ages*** | Noncancer | 292,468 | 2,535 | 1.70 | 1(Ref.) | 1(Ref.) | 1(Ref.) | 1(Ref.) |
|  | Breast cancer | 72,560 | 725 | 1.98 | 1.17 (1.08-1.27) | 1.18 (1.09-1.28) | 1.11 (1.02-1.21) | 1.10(1.01-1.19) |
| ***18-39*** | Noncancer | 15,347 | 16 | 0.20 | 1(Ref.) | 1(Ref.) | 1(Ref.) | 1(Ref.) |
|  | Breast cancer | 3,608 | 13 | 0.71 | 3.59 (1.73-7.45) | 3.57 (1.72-7.41) | 3.40 (1.64-7.08) | 3.44 (1.66-7.16) |
| ***40-50*** | Noncancer | 102,950 | 302 | 0.57 | 1(Ref.) | 1(Ref.) | 1(Ref.) | 1(Ref.) |
|  | Breast cancer | 26,232 | 96 | 0.71 | 1.26 (1.00-1.58) | 1.27 (1.01-1.60) | 1.21 (0.96-1.52) | 1.22 (0.97-1.53) |
| ***51-65*** | Noncancer | 138,354 | 1,146 | 1.62 | 1(Ref.) | 1(Ref.) | 1(Ref.) | 1(Ref.) |
|  | Breast cancer | 33,831 | 352 | 2.07 | 1.28 (1.13-1.44) | 1.30 (1.15-1.46) | 1.22 (1.08-1.38) | 1.21 (1.07-1.37) |
| ***≥66*** | Noncancer | 35,817 | 1,071 | 6.17 | 1(Ref.) | 1(Ref.) | 1(Ref.) | 1(Ref.) |
|  | Breast cancer | 8,889 | 264 | 6.21 | 1.01 (0.88-1.15) | 1.00 (0.88-1.15) | 0.94 (0.82-1.08) | 0.92 (0.80-1.05) |
| P for interaction | |  |  |  | 0.001 | 0.001 | 0.001 | <0.001 |

IR, incidence rate; PYs, person-years; HR, hazard ratio; sHR, sub-distribution hazard ratio; CI, confidence interval.

Model 2 was adjusted for age, income, and area of residence. Model 3 was adjusted for age, income, area of residence, hypertension, diabetes mellitus, dyslipidemia, coronary heart disease, congestive heart failure, chronic kidney disease, and chronic obstructive pulmonary disease. Model 4 was additionally adjusted for body mass index, smoking status, alcohol consumption and regular physical activity from Model 3.

**Table S10.** Adjusted sub-distribution hazard ratios for developing atrial fibrillation by cancer treatment type among breast cancer surgery survivors: **A subset analysis based on data comprising participants in the general health screening examination**

| **Treatment type** | | Subjects (N) | Case (n) | IR | Model 1 (Crude)  HR (95% CI) | Model 2  sHR (95% CI) | Model 3  sHR (95% CI) | Model 4  sHR (95% CI) | Model 5  sHR (95% CI) |
| --- | --- | --- | --- | --- | --- | --- | --- | --- | --- |
| ***Anthracycline*** | No | 36,492 | 369 | 2.03 | 1(Ref.) | 1(Ref.) | 1(Ref.) | 1(Ref.) | 1(Ref.) |
|  | Yes | 36,068 | 356 | 1.94 | 0.95 (0.82-1.10) | 1.41 (1.21-1.64) | 1.37 (1.18-1.60) | 1.57 (1.21-2.03) | 1.55 (1.20-2.01) |
| ***Taxane*** | No | 31,595 | 325 | 2.06 | 1(Ref.) | 1(Ref.) | 1(Ref.) | 1(Ref.) | 1(Ref.) |
|  | Yes | 40,965 | 400 | 1.93 | 0.94 (0.81-1.08) | 1.24 (1.06-1.44) | 1.19 (1.03-1.39) | 0.83 (0.65-1.08) | 0.83 (0.64-1.07) |
| ***Trastuzumab*** | No | 61,922 | 617 | 1.96 | 1(Ref.) | 1(Ref.) | 1(Ref.) | 1(Ref.) | 1(Ref.) |
|  | Yes | 10,638 | 108 | 2.09 | 1.07 (0.87-1.32) | 1.17 (0.95-1.43) | 1.11 (0.90-1.36) | 0.95 (0.76-1.19) | 0.95 (0.77-1.19) |
| ***Endocrine treatment*** | No | 22,012 | 239 | 2.21 | 1(Ref.) | 1(Ref.) | 1(Ref.) | 1(Ref.) | 1(Ref.) |
|  | Tamoxifen | 30,313 | 173 | 1.10 | 0.50(0.41-0.61) | 0.78 (0.64-0.96) | 0.78 (0.64-0.95) | 0.81 (0.66-0.99) | 0.81 (0.66-0.99) |
|  | AIs | 19,317 | 302 | 3.14 | 1.42 (1.20-1.69) | 0.98 (0.82-1.16) | 0.97 (0.82-1.15) | 0.98 (0.82-1.17) | 0.96 (0.81-1.15) |
|  | Both | 918 | 11 | 2.39 | 1.08 (0.59-1.98) | 0.78 (0.43-1.43) | 0.73 (0.40-1.33) | 0.75 (0.41-1.37) | 0.74 (0.40-1.35) |
| ***Radiation treatment*** | No | 20,973 | 257 | 2.47 | 1(Ref.) | 1(Ref.) | 1(Ref.) | 1(Ref.) | 1(Ref.) |
|  | Yes | 51,587 | 468 | 1.79 | 0.73 (0.62-0.85) | 0.97 (0.83-1.14) | 0.99 (0.85-1.16) | 0.96 (0.82-1.12) | 0.95 (0.81-1.11) |

IR, incidence rate (described as per 1,000 person-years); HR, hazard ratio; sHR, sub-distribution hazard ratio; CI, confidence interval; AIs, aromatase inhibitors.

Model 2 was adjusted for age, income, and area of residence. Model 3 was adjusted for age, income, area of residence, hypertension, diabetes mellitus, dyslipidemia, coronary heart disease, congestive heart failure, chronic kidney disease, and chronic obstructive pulmonary disease. Model 4 was additionally adjusted for history of anthracycline, taxane, trastuzumab, endocrine treatment and radiation treatment from Model 3. Model 5 was additionally adjusted for body mass index, smoking status, alcohol consumption and regular physical activity from Model 4.
